# Measurement of Force Required for Anterior Displacement of Intraocular Lenses and Its Defining Parameters

**DOI:** 10.3390/ma13204593

**Published:** 2020-10-15

**Authors:** Mihoko Mochiji, Sachiko Kaidzu, Yoshihisa Ishiba, Yuji Matsuda, Masaki Tanito

**Affiliations:** 1 Department of Ophthalmology, Shimane University Faculty of Medicine, Izumo 693-8501, Japan; kozakana@med.shimane-u.ac.jp (M.M.); kecha@med.shimane-u.ac.jp (S.K.); 2 Technology Development Department, Yamamoto Kogaku Co. Ltd., Higashi-Osaka 577-0056, Japan; ishiba@yamamoto-kogaku.co.jp; 3 Medical Division, Hoya Corporation, Tokyo 160-8347, Japan; yuji.matsuda@hoya.com

**Keywords:** intraocular lens (IOL), one-piece IOL, soft-acrylic IOL, IOL displacement force, IOL hardness, haptics junction area, posterior IOL bulge

## Abstract

Intraocular stability during or after cataract and glaucoma filtration surgeries and vitreous surgery with a gas/silicone oil tamponade might differ among intraocular lenses (IOLs). We used six different one-piece IOL models and measured the force that displaced the IOLs from the vitreous cavity to anterior chamber as a measure of stability against the pressure gradient between the anterior and posterior IOL surfaces. We measured IOL hardness, haptics junction area, and posterior IOL bulge to identify what determines the IOL displacement force. The KOWA YP2.2 IOL (1.231 mN) required significantly greater force than the HOYA XY1 (0.416 mN, *p* = 0.0004), HOYA 255 (0.409 mN, *p* = 0.0003), Alcon SN60WF (0.507 mN, *p* = 0.0010), and Nidek NS60YG (0.778 mN, *p* = 0.0186) IOLs; J&J ZCB00V IOL (1.029 mN) required greater force than the HOYA XY1 (*p* = 0.0032) and HOYA 255 (*p* = 0.0029) IOLs; the Nidek NS60YG IOL required greater force than the HOYA 255 (*p* = 0.0468) IOL. The haptics junction area was correlated positively with the IOL displacement force (*r* = 0.8536, *p* = 0.0306); the correlations of the other parameters were non-significant. After adjusting for any confounding effects, the haptics junction area was correlated significantly with the IOL displacement force (*p* = 0.0394); the IOL hardness (*p* = 0.0573) and posterior IOL bulge (*p* = 0.0938) were not. The forces that displace IOLs anteriorly differed among one-piece soft-acrylic IOLs, and the optics/haptics junction area was the major force determinant.

## 1. Introduction

Intraocular lens (IOL) materials and performance have changed dramatically over the last 30 years [1,2]. The previous mainstream polymethylmethacrylate (PMMA) IOLs have been abandoned in favor of the foldable IOLs along with the changes in the surgical procedures from extracapsular cataract extraction to small-incisional phacoemulsification and aspiration. Soft-acrylic IOLs that entered the market in the early 1990s are the major IOL material used currently because of the high index of refraction and optics that unfold slowly and in a controlled manner [1,2,3,4,5,6,7]. The roles of IOL design on the development of posterior capsular opacity (PCO), the most frequent complication associated with decreased vision after modern cataract surgery, has been studied extensively [4,5,8,9,10,11,12,13]. In an animal study, a sharp capsular bend and capsular adhesion created by a rectangular sharp-edged optic design of the acrylic IOL were associated with the inhibition of lens epithelial cells proliferation [12]. Accordingly, current one-piece soft-acrylic IOLs have adopted the sharp-edge design to achieve a sharper capsular bend, prompt capsular adhesion, and a greater PCO preventive effect [8,10,11,12,13]. Cataract surgery involving the implantation of clear IOLs has increased light transmittance at approximately 410 nm that might be associated with the risk of age-related macular degeneration [14]. To minimize this, yellow-tinted IOLs that absorb blue light are available [3,14,15,16,17,18,19]. A bulky haptic, such as those of the foldable one-piece IOLs can hamper capsular bend formation and adhesion [13,20,21,22,23]. Thin haptics such as those of three-piece acrylic IOLs promote capsular adhesion and might be associated with a lower incidence of PCO than one-piece IOLs [20,21,22,23]. However, the current designs of one-piece IOLs are associated with significantly less anterior capsule opacification and less dysphotopsia than three-piece IOLs [23]. Haptic angulation may have a greater effect on the amount and scatter of postoperative IOL movement and resulting deviations from the calculated target refraction [24]. Thus, one-piece, yellow-tinted, soft-acrylic IOLs with a sharp-edge design and bulky non- or small-angulated haptics have become popular IOLs used during current cataract surgeries.

During or immediately after cataract and glaucoma filtration surgeries and vitreous surgery with a gas or silicone oil tamponade, even inserted into the capsular bag, the IOL position is likely to become unstable due to the pressure difference between the anterior chamber and vitreous cavity; the degree of the instability might differ among IOL models. An unstable position of the IOL can associate with refractive fluctuation and a shallow anterior chamber depth that may lead to corneal endothelial damage. Although the current one-piece IOLs are similar at a glance, the details of their designs and material hardness differ. In the current study, we used six different one-piece IOL models and measured the force required to displace the IOLs from the vitreous cavity side to the anterior chamber side as a measure of stability against the pressure gradient between the front and back of the IOL. We also performed three measurements to elucidate the factors that determine the IOL displacement force.

## 2. Materials and Methods

### 2.1. IOLs 

**Figure 1:**
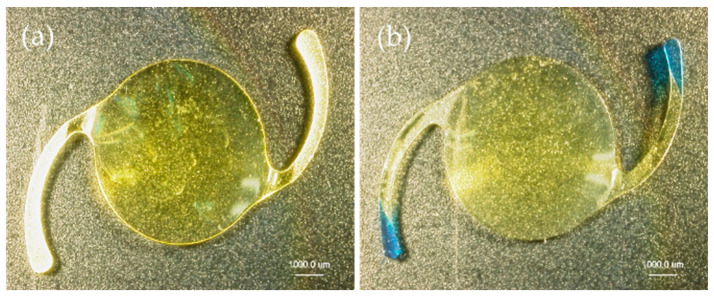

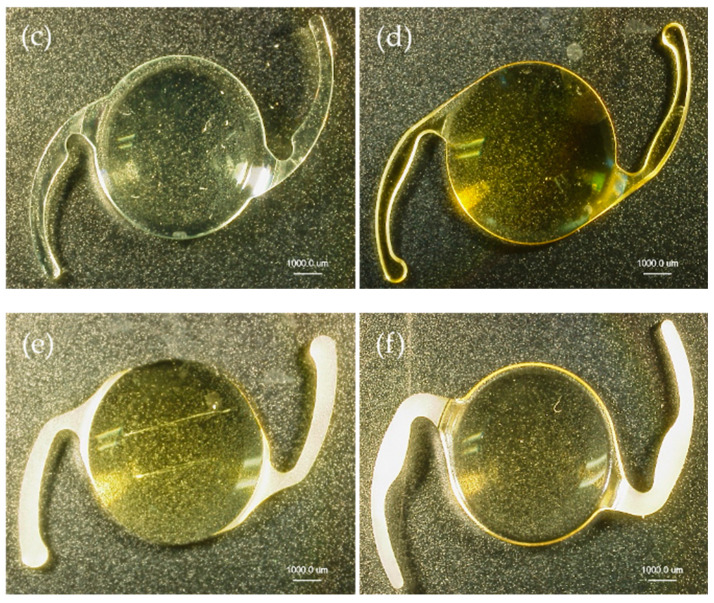
The six Intraocular lens (IOL) models used in this experiment. All are one-piece, soft-acrylic, +20-D refractive force, 6-mm optics diameter. (**a**) HOYA XY-1, (**b**) HOYA 255, (**c**) J&J ZCB00V, (**d**) Alcon SN60WF, (**e**) KOWA YP2.2, and (**f**) Nidek NS60YG.

In this study, 18 IOLs representing six IOL models (*n* = 3 of each model; HOYA 255, HOYA XY1, J&J ZCB00V, Alcon SN60WF, KOWA YP2.2, and Nidek NS60YG) were used (Figure 1, Table 1). All IOL models were single-piece, soft-acrylic, with a 6.0 mm optic diameter and +20 diopter (D) power. The HOYA 255 had a 12.5 mm diameter and a 5° haptic angle. The other five IOL models had 13 mm diameters and 0° haptic angles.

### 2.2. Experimental Settings

#### 2.2.1. IOL Displacement Force

**Figure 2:**
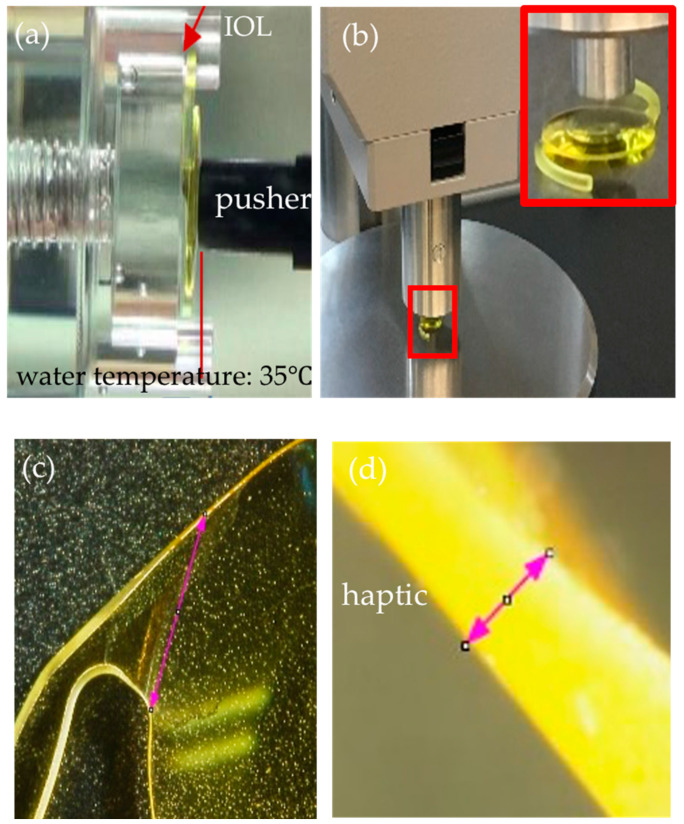

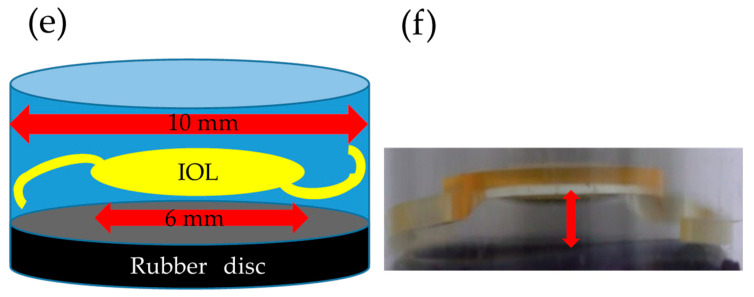
Experimental settings. (**a**) Setting for the IOL displacement force measurement. The forces required for anterior displacement of the IOLs. The IOLs set in the fixture are displaced anteriorly (anterior chamber side) by a pusher from the posterior (vitreous cavity) side until the IOL optics move 1 mm in the water. The water temperature is set at 35 °C. (**b**) The setting for the IOL hardness measurement. The IOL is indented by the indenter perpendicular to the center of the optics. (**c**,**d**) The settings for the string lengths (**c**, pink double arrow) and thickness (**d**, pink double arrow) measurements of the haptics/optics junction area. (**e**,**f**) The settings for the posterior IOL bulge measurement. The IOLs are set in a clear cylinder with an inner diameter of 10 mm (**e**), and the distance between the rubber disc surface and surface of the IOL (red double arrow) is measured.

To estimate the intraocular stability of an IOL against its anterior displacing effect from the vitreous cavity, the force required to anteriorly displace the IOLs was measured. The IOLs were set in a fixture (diameter, 10 mm) and immersed in water at a temperature of 35 °C (Figure 2a). The IOLs were pushed from the posterior side (i.e., vitreous side) by a pusher (diameter, 3 mm) until they moved 1 mm anteriorly (i.e., anterior chamber side). The force required for anterior IOL displacement was monitored using a micro-load measuring system (Sankyo International Co., Tokyo, Japan) connected to a pusher at 0.1 mm increment steps. The measured data are expressed in mN.

#### 2.2.2. IOL Hardness

The IOL hardness was measured using the automatic hardness tester (Digi test II, Bareiss Prüfgerätebau GmbH, Oberdischingen, Germany) at room temperature (Figure 2b). The IOLs were pushed vertically with constant pressure by an indenter for 30 s, the IOLs were released, and the amount of rebound of the IOL material was measured for 5 s. The measured value is expressed in international rubber hardness degrees (IRHDs).

#### 2.2.3. Haptics Junction Area

Digitized photographs of the haptics/optics junction area were obtained using a multi-angle stereo microscope and digital camera system (VB-7010/VB-G25, Keyence Co. Ltd., Osaka, Japan) from two angles (Figure 2c,d). Using the equipped software, the string length (Figure 2c, pink double arrow) and thickness (Figure 2d, pink double arrow) were measured at the haptics/optics junction. The arc length of the junction was calculated from the measured string length and IOL radius of 3 mm, and the area of the haptics junction was estimated by multiplying the arc length and lens thickness.

#### 2.2.4. Posterior IOL Bulge

The IOL was set in a clear cylinder with a 10-mm inner diameter according to the posterior side (i.e., vitreous side) of the IOL is being upside (Figure 2e). The digitized pictures then were obtained using a multi-angle stereo microscope system (VB-7010/VB-G25, Keyence Co. Ltd., Osaka, Japan) from a side of the IOL, and the distance from the bottom of the cylinder and anterior surface (i.e., anterior chamber side) was measured as the posterior IOL bulge (Figure 2f, red double arrow).

### 2.3. Statistical Analysis 

The experiments were repeated using three different IOLs of each IOL model. The continuous data are expressed as the means ± standard deviations. Statistical analyses were performed using JMP version 14.2 software (JMP Statistical Discovery, Cary, NC, USA). The mean force required for IOL displacement from the 0.0 mm position to the 1.0 mm position was compared between each pair of IOL models using an unpaired *t*-test. The correlations between the IOL displacement force and the three estimated parameters were assessed by linear regression models. The correlations also were assessed using a mixed-effects regression model with three estimated parameters (i.e., IOL hardness, haptics junction area, and posterior IOL bulge), which were set as a fixed effect and the IOL models were set as a random effect. *P* < 0.05 was considered statistically significant.

## 3. Results

**Figure 3:**
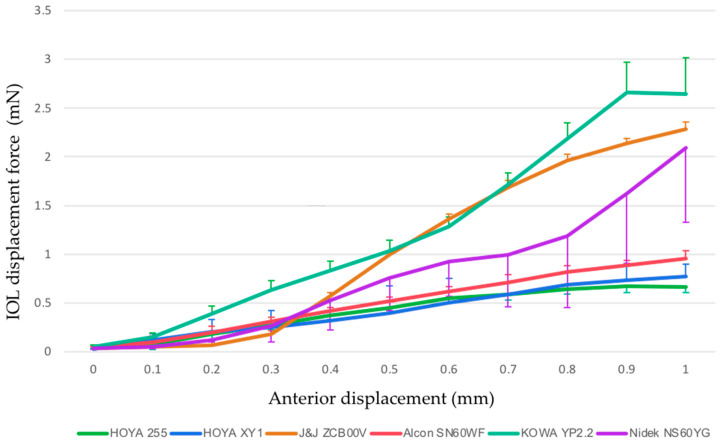
Changes in the IOL displacement force from the 0 and 1 mm points in each IOL model. Error bar, standard deviation.

The IOL displacement forces of each model are summarized in Figure 3 and Table 2. The statistical comparisons (Table 3) showed that the KOWA YP2.2 IOL (1.231 mN) required significantly greater force than the HOYA XY1 (0.416 mN, *p* = 0.0004), HOYA 255 (0.409 mN, *p* = 0.0003), Alcon SN60WF (0.507 mN, *p* = 0.0010), and Nidek NS60YG (0.778 mN, *p* = 0.0186) IOLs; the J&J ZCB00V IOL (1.029 mN) required greater force than the HOYA XY1 (*p* = 0.0032) and HOYA 255 (*p* = 0.0029) IOLs; the Nidek NS60YG IOL required greater force than the HOYA 255 IOL (*p* = 0.0468) (Table 3). 

**Figure 4:**
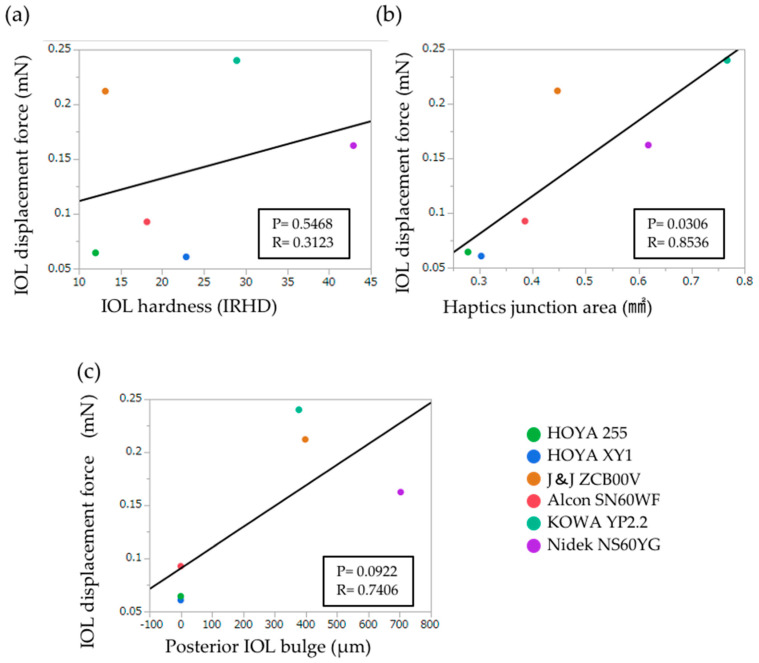
Correlation between the IOL displacement force and IOL hardness (**a**), haptics junction area (**b**), and posterior IOL bulge (**c**). *P* values and regression coefficient (*R*) are calculated by linear regression analyses.

To assess the factors that may determine the IOL displacement force, we predicted three parameters—i.e., IOL hardness, haptics junction area, and posterior IOL bulge; we estimated them in each IOL model (Figure 4). Using a linear regression model, the haptics junction area was correlated positively with the IOL displacement force (*r* = 0.8536, *p* = 0.0306), while the correlations of the other two parameters with the IOL displacement force were not significant (Figure 4). After adjusting for confounding effects among the three parameters using a mixed-effects regression model, the haptics junction area again was correlated significantly with the IOL displacement force (*p* = 0.0394), while the IOL hardness (*p* = 0.0573) and posterior IOL bulge (*p* = 0.0938) remained statistically non-significant (Table 4).

## 4. Discussion

We measured the force required for the anterior displacement of IOLs and its three defining parameters among the currently available major IOL models. The results showed that the forces differed markedly depending on each IOL model, and the haptics junction area was the major determinant of the force. Since the introduction of IOLs, their intraocular stability has been discussed [25]. In clinical settings, cases in which the microscopic anterior or posterior displacement of IOLs accompanying myopic or hyperopic shifts from the expected are seen. In previous studies, acrylic IOLs are characterized by less anterior capsule contraction and less or the same decentration compared with PMMA and silicone IOLs [25,26,27,28,29]. Although some studies have investigated anterior chamber depth in eyes before and after IOL implantation [30,31,32,33], the measurements of the biomechanical characteristics of the IOLs based on the anterior displacement force of the IOL are unique in the literature.

The anterior displacement force of the KOWA YP2.2 IOL (1.231 mN) was three times higher than that of the HOYA 255 IOL (0.409 mN); thus, the forces are widely distributed. Although the hardness values of the HOYA 255 (12.0 IRHD) and XY1 (22.9 IRHD) IOLs differed by a factor of 2, the anterior displacement forces of these IOLs were equivalent; the Nidek NS60YG (42.9 IRHD) was by far the hardest IOL but did not require the greatest force. Thus, the IOL hardness is unlikely the sole determinant of the IOL displacement force. The posterior IOL bulge in our study resembles, but is different from, the International Organization for Standardization (ISO) 11979-3 specified “measurement of axial displacement in compression”, which measures the axial displacement distance before and after the compression [34]. To the best of our knowledge, the posterior IOL bulge and junction area have not been described previously as major parameters of the IOL design. The IOL bulge was greatest with the Nidek NS60YG IOL (703 µm) but was 0 µm with the HOYA XY1, HOYA 255, and Alcon SN60WF IOLs. The IOL size and haptic angle did not differ greatly among these IOLs; thus, factors other than the IOL size and haptic angle affect the amount of posterior IOL bulge, but these remain to be elucidated. The current results suggested that the IOL bulge is not the sole determinant of the IOL displacement force. The haptics junction area of the KOWA YP2.2 IOL (0.879 mm^2^) is three times greater than that of HOYA XY1 (0.289 mm^2^) and HIOYA 255 (0.319 mm^2^); the distributions of the haptics junction areas were well correlated with the distribution of the IOL displacement forces (Figure 4b); thus, the results indicate that the anterior displacement forces can be predicted by the IOL junction areas. A recent study has revealed that ISO 11979-3 specified that “IOL compression force” [34] is affected by the width and thickness of the haptics [35]; accordingly, the haptic itself and haptics–optics junction seem to have important roles in the stability of IOL.

We used six IOL models with three IOLs for each IOL model in this experiment. We cannot exclude the possibility that the roles of IOL hardness and bulge become statistically significant if we increase the number of samples and/or IOL models in the experiments. When we measured the IOL bulge, setting the IOLs in a cylinder with a 10 mm diameter required several trials; thus, possible IOL damage during the setting process due to pinching of the IOLs several times might have affected the results. It is important to note that the IOL displacement force is the measure of the biomechanical features of IOLs; thus, the clinical significance of its role remains to be clarified.

## 5. Conclusions

In conclusion, the forces required to displace IOLs anteriorly differed among modern one-piece soft-acrylic IOLs, and the optics–haptics junction area is a major determinant of the force.

## Figures and Tables

**Table 1:** Summary of IOL models.

IOL Model	Optic Design	Optical Material	Refractive Power (D)	Refractive Index	IOL Diameter (mm)	Optic Diameter (mm)	Haptics Angle (°)	Optic Thickness (mm)	Haptic Thickness (mm)
HOYA XY1	Aspherical Lens	Acrylic Resin	+20	1.55	13.0	6	0	0.58	0.4
HOYA 255	Aspherical Lens	Acrylic Resin	+20	1.52	12.5	6	5	0.66	0.4
J&J ZCB00V	Aspherical Lens	Acrylic Resin	+20	1.47	13.0	6	0	0.72	0.49
Alcon SN60WF	Aspherical Lens	Acrylic Resin	+20	1.55	13.0	6	0	0.59	0.43
KOWA YP2.2	Aspherical Lens	Acrylic Resin	+20	1.52	13.0	6	0	0.7	0.35

**Table 2:** Forces required for anterior displacement of each IOL model (mN).

IOL Model	Anterior Displacement (mm)											
	0.0	0.1	0.2	0.3	0.4	0.5	0.6	0.7	0.8	0.9	1.0	Mean
HOYA 255	0.036 ± 0.017	0.075 ± 0.051	0.177 ± 0.064	0.275 ± 0.049	0.373 ± 0.042	0.448 ± 0.026	0.546 ± 0.047	0.588 ± 0.056	0.641 ± 0.052	0.673 ± 0.064	0.664 ± 0.061	0.409 ± 0.236
HOYA XY1	0.023 ± 0.009	0.118 ± 0.066	0.203 ± 0.128	0.248 ± 0.176	0.317 ± 0.22	0.392 ± 0.281	0.5 ± 0.252	0.585 ± 0.203	0.686 ± 0.196	0.729 ± 0.161	0.771 ± 0.126	0.416 ± 0.309
J&J ZCB00V	0.036 ± 0.009	0.052 ± 0.012	0.065 ± 0.017	0.177 ± 0.008	0.572 ± 0.059	0.99 ± 0.037	1.36 ± 0.059	1.683 ± 0.073	1.958 ± 0.092	2.141 ± 0.094	2.282 ± 0.125	1.029 ± 0.869
Alcon SN60WF	0.042 ± 0.012	0.095 ± 0.067	0.199 ± 0.061	0.311 ± 0.041	0.415 ± 0.036	0.52 ± 0.042	0.615 ± 0.052	0.713 ± 0.074	0.814 ± 0.07	0.889 ± 0.046	0.958 ± 0.076	0.507 ± 0.314
KOWA YP2.2	0.049 ± 0.021	0.147 ± 0.042	0.389 ± 0.082	0.634 ± 0.094	0.834 ± 0.092	1.03 ± 0.111	1.288 ± 0.096	1.713 ± 0.121	2.18 ± 0.168	2.658 ± 0.315	2.641 ± 0.376	1.231 ± 0.938
Nidek NS60YG	0.033 ± 0.018	0.052 ± 0.024	0.118 ± 0.021	0.262 ± 0.161	0.523 ± 0.3	0.758 ± 0.334	0.925 ± 0.355	0.99 ± 0.531	1.183 ± 0.727	1.625 ± 0.716	2.092 ± 0.764	0.778 ± 0.798

The data are expressed as the mean ± standard deviation.

**Table 3:** Difference in forces required for anterior IOL displacement among the various IOL models.

IOL Model	HOYA XY1	HOYA 255	J&J ZCB00V	Alcon SN60WF	KOWA YP2.2	Nidek NS60YG
HOYA XY1	–	Dif = 0.008	−0.613	−0.091	−0.815	−0.361
HOYA 255	*p* = 0.9638	–	−0.621	−0.098	−0.822	−0.369
J&J ZCB00V	0.0032	0.0029	–	0.522	−0.202	0.252
Alcon SN60WF	0.5964	0.5659	0.0086	–	−0.724	−0.271
KOWA YP2.2	0.0004	0.0003	0.2498	0.0010	–	0.453
Nidek NS60YG	0.0509	0.0468	0.1567	0.1301	0.0186	–

The forces required for anterior IOL movement from 0.0 to 1.0 mm shown in Table 1 are averaged for each IOL model. Dif (right upper cells) = least-squares mean difference between each pair of IOLs (mm); p (left lower cells) = *p* values for the comparison between each pair of IOLs using the unpaired *t*-test.

**Table 4:** IOL hardness, haptics junction area, and posterior IOL bulge in each IOL model.

IOL Models	HOYA XY1	HOYA 255	J&J ZCB00V	Alcon SF60WF	KOWA YP2.2	Nidek NS60YG	Estimate	*p* Value
IOL Hardness (IRHD)	22.9 ± 0.3	12.0 ± 0.3	13.2 ± 0.1	18.2 ± 0.2	28.9 ± 0.4	42.9 ± 0.6	−0.0046	0.0573
Haptics Junction Area (mm^2^)	0.289 ± 0.013	0.319 ± 0.013	0.512 ± 0.036	0.443 ± 0.046	0.879 ± 0.033	0.706 ± 0.086	0.3444	0.0394
Posterior IOL Bulge (µm)	0	0	398 ± 138	0	378 ± 70	703 ± 109	0.0002	0.0938

Estimate and *p* values are calculated using the mixed-effects regression model.
